# Analysis of the Significance of Changes in the Number and Energy Parameters of Acoustic Emission Signals on the Assessment of the Strength of Fibre–Cement Boards

**DOI:** 10.3390/ma15165757

**Published:** 2022-08-20

**Authors:** Anna Adamczak-Bugno, Grzegorz Świt, Aleksandra Krampikowska, Edoardo Proverbio

**Affiliations:** 1 Faculty of Civil Engineering and Architecture, Kielce University of Technology, 25-314 Kielce, Poland; 2 Department of Engineering, University of Messina, Contrada di Dio Sant’Agata, 98166 Messina, Italy

**Keywords:** fibre–cement composites, acoustic emission method, statistical analysis

## Abstract

The article presents the results of three-point bending tests carried out for samples cut from full-size fibre–cement boards subjected to typical and exceptional conditions. The tests were carried out with the simultaneous acquisition of acoustic emission signals. It has been noted that some factors significantly deteriorate the strength parameters of the samples as well as cause the occurrence of differences in the number of acoustic emission signals of various classes and their energy parameters. A statistical analysis was carried out in order to repeat the relationship between the strength parameters of the samples and the acoustic emission parameters. Based on the research, it was found that the *MOR* bending strength for specimens exposed to fire and high temperature is more than 50% lower than for air-dried specimens and specimens exposed to water. The increased number of freeze–thaw cycles also has an impact on the strength of the specimens. Components exposed to more than 10 freeze–thaw cycles had a strength more than 30% smaller than the reference specimens soaked in water and exposed to bath-drying cycles. A similar dependency was indicated by the number of signals of the individual classes, their energy parameters and their frequencies. The number, strength, duration and frequency also decreased along with the increase in the test case number. On this basis, conclusions were drawn concerning the suitability of acoustic emission for the evaluation of the strength of fibre–cement elements.

## 1. Introduction

### 1.1. Description of Fibre–Cement Boards

Fibre cement (Lat. fibro–fibre and caementum–cement) is a construction material consisting of cement or calcium silicate (formed by the chemical reaction of silicate and calcium materials) and mineral fillers, reinforced with fibres (randomly dispersed or continuous strands and tapes or meshes and fabrics). Fibre–cement boards have high mechanical strength (the minimum bending strength of flat cement–fibre boards for wall cladding is 4 MPa), flexibility and durability [1,2,3,4,5].

Contemporary fibre–cement products (sold legally on the EU market) are harmless to people. In most cases, fibre–cement cladding is resistant to corrosion, rot and fungi as well as UV radiation.

Fibre–cement boards are manufactured using pressing and autoclaves. The pressing pressure is approximately 650 N/cm^2^. After the pressing cycle, the boards pass the curing stage within 6–8 h and are then placed in autoclaves, where they finally harden at a high temperature of 175 °C and under the pressure of 10 atm. Thanks to this technology, fibre–cement boards have high mechanical strength and bending strength. Some manufacturers of fibre–cement cladding use recycled materials during production [5,6,7,8].

The appearance of fibre–cement cladding may vary owing to the virtually limitless options regarding colour, texture and size. This fact, combined with the technical and functional performance, is the reason the use of fibre–cement rainscreen cladding is not limited by regional architectural traditions, climatic conditions or intended use of the building. Fibre–cement cladding may be full-body coloured, made with an outer textured layer (imitating wood or stone), painted after installation or even covered with render. That is why rainscreen cladding using fibre–cement cladding can be found on new and reconstructed buildings of any type and with any function (apartment buildings and single-family houses, office buildings, industrial facilities, hospitals, etc.) [9,10,11,12].

End-of-life fibre–cement cladding can be recycled.

Initially, fibre–cement products were made using asbestos. The first prototypes of fibre–cement boards were manufactured towards the end of the 19th century by Ludwig Hatschek from Austria as a mixture of cement and asbestos. Around the end of the 19th century, there was a need for materials that would be less flammable, less expensive, more resistant to variable temperatures, stronger and more durable than the conventional construction materials known at the time. The first Austro-Hungarian asbestos factory (owned by Ludwig Hatschek) manufactured fibre–cement roof cladding. The production of wall cladding followed soon after.

By 1976, fibre–cement cladding was mostly made using asbestos. After it was established that asbestos was harmful to people, approx. 200 types of fibres (e.g., basalt, cellulose) were started to be used to make fibre–cement cladding [7,10,13].

### 1.2. Application and Operating Conditions of Fibre–Cement Boards

Fibre–cement cladding is an essential component of rainscreen cladding, which is growing increasingly popular both with designers and clients. The popularity of such facades is owed to aesthetic considerations (a very wide range of exterior cladding that enables the adaptation of building facades to any urban environment), ease of installation, competitive prices, thermal insulation performance and inexpensive maintenance. Rainscreen cladding is used on all types of buildings with various functions, fit-out specifications and furnishings. Such buildings include large shopping centres, sports and recreational facilities, railway stations, high-end high-rise office buildings, small single-family houses, etc. [14,15].

Rainscreen cladding is increasingly popular with clients and contractors and is becoming more widespread. Unfortunately, this increases the number of observed failures of such solutions.

Typical rainscreen cladding faults include the separation of the cladding from the support framework, which is particularly dangerous to people and property if it occurs at a large height.

Rainscreen cladding has very high durability and reliability, but it requires continuous monitoring to correctly assess its condition. The absence of monitoring and correct diagnostics of rainscreen cladding often causes it to wear out and suffer damage.

Rainscreen cladding, regardless of the materials it is made of, has varying resistance to operating conditions. The most aggressive factors that adversely affect the safety of use of wall-cladding systems include climatic and environmental factors and anthropogenic (human-related) factors [16,17,18,19].

Climatic and environmental factors include:

- Temperature variations

During use, exterior rainscreen cladding is exposed to significant temperature variations (daily and seasonal), which cause stresses and linear strain in the cladding due to thermal expansion. Temperature variations during the day may reach more than 40 °C, and they often cause the cladding to crack and separate from the support framework (in the case of adhesive cladding).

- Thermal shock

Fibre–cement cladding used as rainscreen cladding is exposed to thermal shocks, i.e., sudden temperature variations during abrupt weather changes. Dark-coloured cladding may heat up to more than 90 °C during the summer season. When torrential rains start (the water temperature may be approx. 10 °C), the exterior parts of the cladding are cooled immediately. The high difference between the temperatures out-side and inside the cladding often results in the appearance of cracks.

- Impact of moisture on rainscreen cladding (rain and snow on the outside and water vapour migration on the inside)

Changes in moisture content affect the shape and dimensions of the cladding. The unavoidable factors that increase water migration into the cladding system include wind. During rainfall, the suction caused by the wind causes the individual parts of the cladding to move and form corridors for the migration of water into the system. If the plastic vapour barrier is damaged or missing, the thermal insulation may become permanently damp.

- Chemical air pollution and moisture

Serious damage to the cladding, grids and mechanical fasteners may be caused by chemical reactions occurring between the chemical elements in the air (due to industrial and transport pollution, from combined heat and power plants, etc.) and moisture (water). Such reactions occur in situations where chemical elements in the air dissolve in the moisture penetrating into the cladding or settling on the metallic support frames.

- Wind actions

Wind fluctuations cause the appearance of long-time, recurring pushing-pulling load, resulting in progressive damage to “cladding-framework” joints.

The most dangerous anthropogenic actions include impacts. Fibre–cement cladding has a fairly low impact strength. Impact with a hard object (e.g., a rock) or soft object (e.g., a ball used by children to play) may cause the cladding to crack. Even microcracks may allow moisture into the cladding, accelerating its degradation.

These factors have a significant impact on the safety of the use of wall-cladding systems, which is why such systems should be systematically monitored to ensure long and safe use [20,21,22,23].

### 1.3. Diagnostic Methods Used to Monitor Fibre–Cement Cladding

The mechanical assessment of fibre–cement boards after exposure to environmental and accidental operating factors is an important issue both from the scientific and practical perspectives.

So far, the vast majority of research on fibre–cement boards has focused only on determining the normative physical and mechanical parameters. The studies determined the impact of operating conditions on the material, including the cycles of heat and rain exposure, freezing and thawing, water-soaking and drying and high and low temperatures, and the composition was modified primarily with respect to the type of the fibres. The mechanical parameters of the boards were determined primarily by means of bending-strength tests [24].

The most well-covered topic in the literature is mainly the impact of high temperatures on concrete components and related processes [25]. The significance of this accidental factor was often tested using non-destructive methods (NDT), primarily using the ultrasonic method and acoustic emission [22,23].

Acoustic methods are advanced enough to also track the frequencies where the recorded acoustic emission (AE) signals appear by using the Fourier transform and wavelet analysis [24].

The acoustic emission (AE) method is one of the popular methods for monitoring civil engineering structures such as bridges, reinforced concrete and steel structures and members, steel and plastic pipelines, compressed gas tanks, internal combustion engines and power transformers. This procedure belongs to the group of passive methods, meaning that AE devices do not emit any signals and do not affect the physical condition of the object under test, only recording the physical effects appearing spontaneously in the monitored object. The sources of acoustic emission signals include appearing and propagating microcracks, cracks and corrosion processes.

An indirect objective of the research described in this paper was to analyse the changes in the mechanical parameters of fibre–cement boards exposed to the previously mentioned typical and accidental operating conditions. An important part of the testing procedure was to analyse the acoustic emission signals recorded during the loading of material samples. The primary objective of the research, in turn, was to examine the correlation of the mechanical parameters of fibre–cement objects with the parameters of the recorded acoustic emission signals. This enabled the assessment of the suitability of the acoustic emission method as a potential reliable diagnostic tool to assess the condition of rainscreen cladding made of fibre–cement boards [25,26,27,28].

The novelty of the research consists mainly in the use of multicriteria analysis of acoustic emission signals to monitor the condition of fibre–cement panels. The use of dividing the recorded acoustic emission signals into classes is a much more reliable approach than the commonly used inference about the state of various materials based on single parameters. Establishing a statistical correlation between the mechanical and acoustic parameters for panels subjected to typical and exceptional conditions will potentially make it possible to use the acoustic emission method to assess the condition of panels at low load levels.

## 2. Materials and Methods

The analysis of the significance of the correlation between changes in the number and energy parameters of acoustic emission signals and the strength of fibre–cement boards was performed for the following test cases:

- air-dried specimens (F_1_)—reference samples stored under constant laboratory conditions (+23 °C, 60% humidity);
- specimens soaked in water for 1 h (F_2_)—samples immersed in water at room temperature (about 23 °C) for 1 h and subjected to a wet bend test;
- specimens soaked in water for 24 h (F_3_)—samples immersed in water at room temperature (about 23 °C) for 24 h and subjected to a wet bend test;
- specimens after 25 bath-drying cycles (F_4_)—samples immersed in water at an ambient temperature above 5 °C (approximately 23 °C) for 18 h and dried in a ventilated oven at 60 °C (±5 °C), with a relative humidity of less than 20% for 6 h for 25 cycles;
- specimens after 50 bath-drying cycles (F_5_)—samples immersed in water at an ambient temperature above 5 °C (approximately 23 °C) for 18 h and dried in a ventilated oven at 60 °C (±5 °C), with a relative humidity of less than 20% for 6 h for 50 cycles;
- specimens after 10 freeze–thaw cycles (F_6_)—samples frozen in a freezer at a temperature of −20 °C (±2 °C) for 2 h, kept at this temperature for another hour, thawed in a water bath at 20 ℃ (±2 °C) for two hours and kept at this temperature for another hour; repeated for 10 cycles;
- specimens after 25 freeze–thaw cycles (F_7_)—samples frozen in a freezer at a temperature of −20 °C (±2 °C) for 2 h, kept at this temperature for another hour, thawed in a water bath at 20 ℃ (±2 °C) for two hours and kept at this temperature for another hour; repeated for 25 cycles;
- specimens after 50 freeze–thaw cycles (F_8_)—samples frozen in a freezer at a temperature of −20 °C (±2 °C) for 2 h, kept at this temperature for another hour, thawed in a water bath at 20 °C (±2 °C) for two hours and kept at this temperature for another hour; repeated for 50 cycles;
- specimens after 100 freeze–thaw cycles (F_9_)—samples frozen in a freezer at a temperature of −20 °C (±2 °C) for 2 h, kept at this temperature for another hour, thawed in a water bath at 20 °C (±2 °C) for two hours and kept at this temperature for another hour; repeated for 100 cycles;
- specimens exposed to direct flame (heating the material to 400 °C) for 2.5 min (F_10_);
- specimens exposed to direct flame (heating the material to 400 °C) for 5 min (F_11_);
- specimens exposed to direct flame (heating the material to 400 °C) for 7.5 min (F_12_);
- specimens exposed to direct flame (heating the material to 400 °C) for 10 min (F_13_);
- specimens exposed to a temperature of 230 °C for 3 h (F_14_)—samples fired in a laboratory furnace to degrade cellulose fibers.

Each group consisted of 10 samples.

Recipes of fibre–cement boards and their technological process are strictly protected by producers, so information on specific ingredients, their quantities and suppliers as well as production details are severely limited. The tested fibre–cement boards were made with the use of basic components such as: CEM I 42.5N Portland cement, cellulose fibers and lime flour additives. The plates were produced using the Hatschek process.

**Figure 1:**
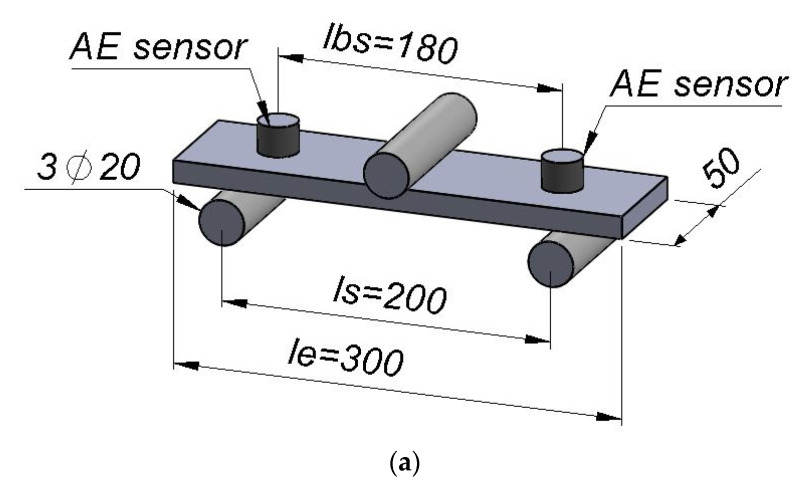

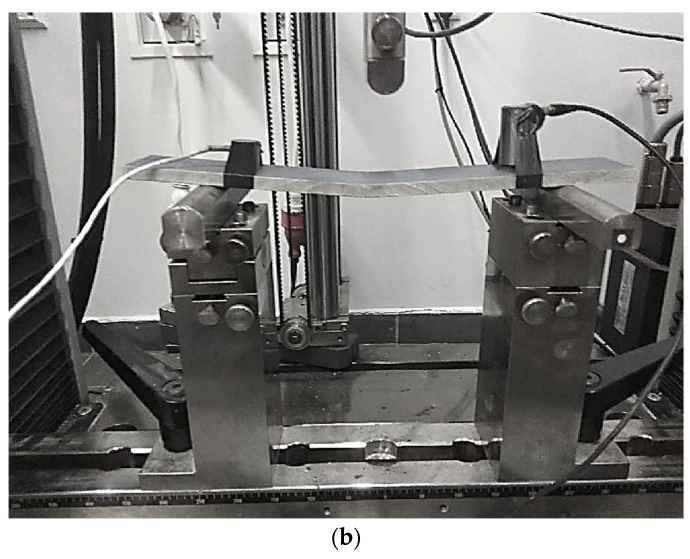
Test stand: (**a**) scheme; (**b**) photograph.

The acoustic emission method was used to assess the development of acoustic emission descriptors depending on the change of the mechanical parameters of fibre–cement boards during a three-point bending test for previously prepared specimens. Figure 1 shows a scheme and photograph of the testing station. Dimensions are given in millimeters. Markings: l_bs_-distance between the axes of sensors, l_s_-distance between the axes of support and l_e_-total length of the sample.

Acoustic emission measurements were performed using two frequency sensors: VS30-SIC with a flat characteristic in the range of 25–80 kHz and VS150-RIC with a measuring range of 100–450 kHz and a peak at 150 kHz. The AEWin procesoor of acoustic emission (Physical Acoustic Corporation, West Windsor, NJ, USA) was used in the tests.

The fibre–cement composite was subject to bending strength tests using the Zwick Roell strength testing machine with a load range from 0 to 10 kN. The tests of the fibre–cement specimens were performed with a constant traverse speed of 0.1 mm/min. The digital signals were processed using Vallen VisualAE (Vallen GMBH, Wolfratshausen, Germany) and Vallen VisualClass (VisualAE (Vallen GMBH, Wolfratshausen, Germany) software for the analysis of AE signals.

## 3. Results

The AE signals recorded during three-point bending tests were broken down into classes using the *k-means* algorithm. The grouping identified four classes that were assigned to the processes occurring in the reinforced cement material under load, based on the authors own prior research and data in the literature [11]:

Class 1—commencement of microcracks;
Class 2—development of a mesh and increase or crack width;
Class 3—delamination of the material and debonding of fibres;
Class 4—breaking and material failure.

The analysis of the test results included the monitoring of the data concerning the number of signals of the individual classes for successive test cases, their strength and duration and average frequencies. The analysis also concerned the strengths achieved for the individual specimens.

The analysis was carried out using IBM SPSS Statistics 26 (IBM, New York, NY, USA). The significance level was adopted as 0.05. The normality of the distributions was verified using the Shapiro–Wilk test, and variance homogeneity was tested using the Levene test. Since there was no normal distribution for some of the data and there was no variance homogeneity in most cases, the average distributions were compared using a group of nonparametric tests for the independent variables, including, in particular, the Kruskal–Wallis test for multiple groups. The dependency between the data was examined using the Spearman’s correlation coefficient due to the absence of normal distribution in the data.

### 3.1. Distribution of the Number and Selected Energy Parameters of Acoustic Emission Signals and Strength for the Individual Test Cases

#### 3.1.1. Number of Class-1 Signals

**Figure 2:**
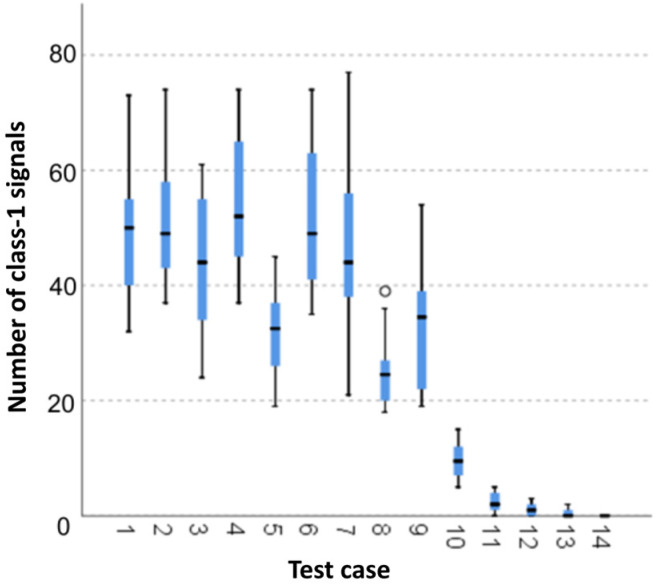
Graphic presentation of the Kruskal–Wallis test results for independent samples: number of class-1 signals.

The graphical representation of Kruskal–Wallis test results for independent samples of the number of class-1 signals (Figure 2) indicates that the highest number of class-1 signals was recorded for components from test case F_7_ (specimens exposed to 25 freeze–thaw cycles). Moreover, for this case, the scatter of the results was the greatest. Test case F_8_ (specimens exposed to 50 freeze–thaw cycles) contains individual outliers. The smallest number of class-1 signals was recorded for case F_14_ (specimens fired in a furnace).

#### 3.1.2. Average Strength of Class-1 Signals [nVs]

**Figure 3:**
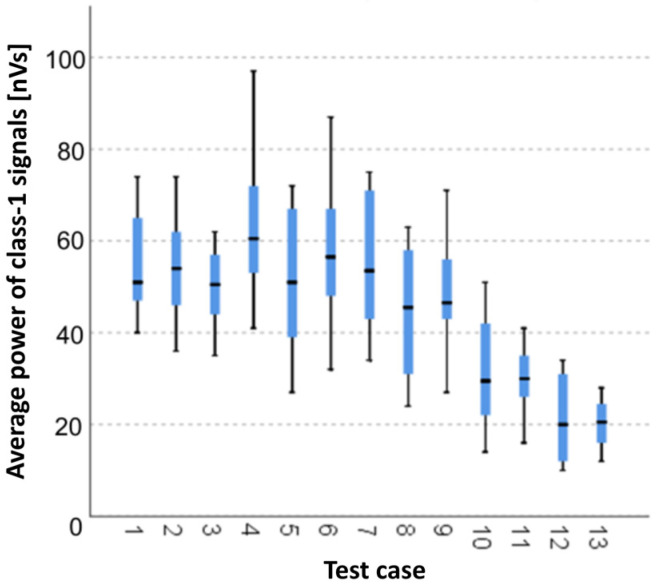
Graphic presentation of the Kruskal–Wallis test results for independent samples: average strength of class-1 signals.

The graphical representation of Kruskal–Wallis test results for independent samples of the average strength of class-1 signals (Figure 3) indicates that the highest strength of class-1 signals was recorded for components from test case F_4_ (specimens exposed to 25 bath-drying cycles). Moreover, for this case, the scatter of the results was the greatest. The smallest average strength of class-1 signals was recorded for case F_12_ (specimens ignited for 7.5 min).

#### 3.1.3. Average Duration of Class-1 Signals [µs]

**Figure 4:**
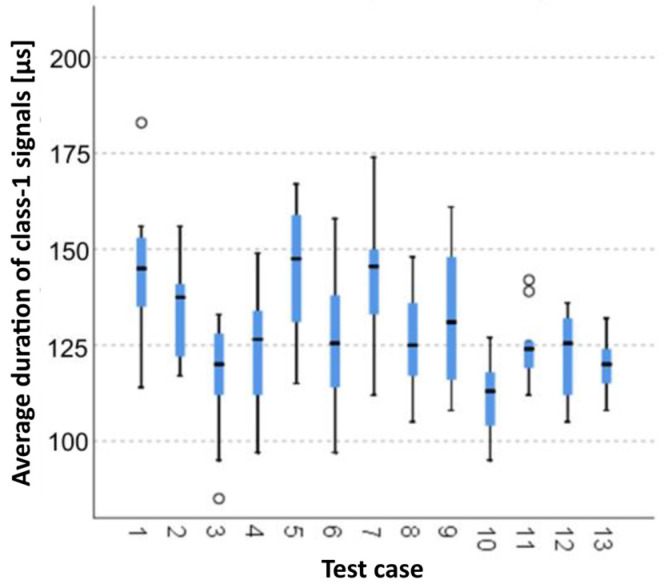
Graphic presentation of the Kruskal–Wallis test results for independent samples: average duration of class-1 signals.

The graphical representation of Kruskal–Wallis test results for independent samples of the average duration of class-1 signals (Figure 4) indicates that the longest average duration of class-1 signals was recorded for components from test case F_7_ (specimens exposed to 25 freeze–thaw cycles). Moreover, for this case, the scatter of the results was the greatest. Test cases F_1_ (air-dried specimens), F_3_ (specimens soaked in water for 24 h) and F_11_ (specimens ignited for 5 min) contain individual outliers. The shortest average duration of class-1 signals was recorded for case F_10_ (specimens ignited for 2.5 min).

#### 3.1.4. Number of Class-2 Signals

**Figure 5:**
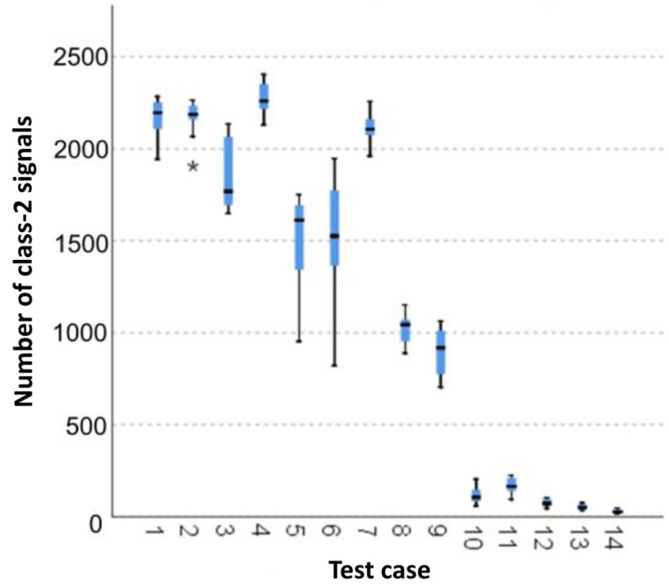
Graphic presentation of the Kruskal–Wallis test results for independent samples: number of class-2 signals.

The graphical representation of Kruskal–Wallis test results for independent samples of the number of class-2 signals (Figure 5) indicates that the highest number of class-2 signals was recorded for components from test case F_4_ (specimens exposed to 25 bath-drying cycles). The largest scatter of results occurred for group F_6_ (specimens exposed to 10 freeze–thaw cycles). Test case F_2_ (specimens soaked in water for 1 h) contains individual outliers. The smallest number of class-2 signals was recorded for case F_14_ (specimens fired in a furnace).

#### 3.1.5. Average Strength of Class-2 Signals [nVs]

**Figure 6:**
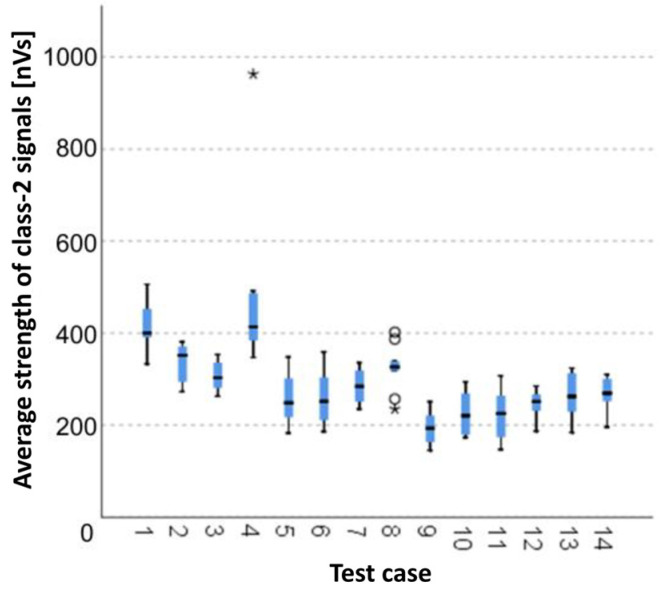
Graphic presentation of the Kruskal–Wallis test results for independent samples: average strength of class-2 signals.

The graphical representation of Kruskal–Wallis test results for independent samples of the average strength of class-2 signals (Figure 6) indicates that the highest average strength of class-2 signals was recorded for components from test case F_1_ (air-dried specimens). Moreover, for this case, the scatter of the results was the greatest. Test cases F_4_ (specimens exposed to 25 bath-drying cycles) and F_8_ (specimens exposed to 50 freeze–thaw cycles) contain individual outliers. The smallest average strength of class-2 signals was recorded for case F_9_ (specimens exposed to 100 freeze–thaw cycles).

#### 3.1.6. Average Duration of Class-2 Signals [µs]

**Figure 7:**
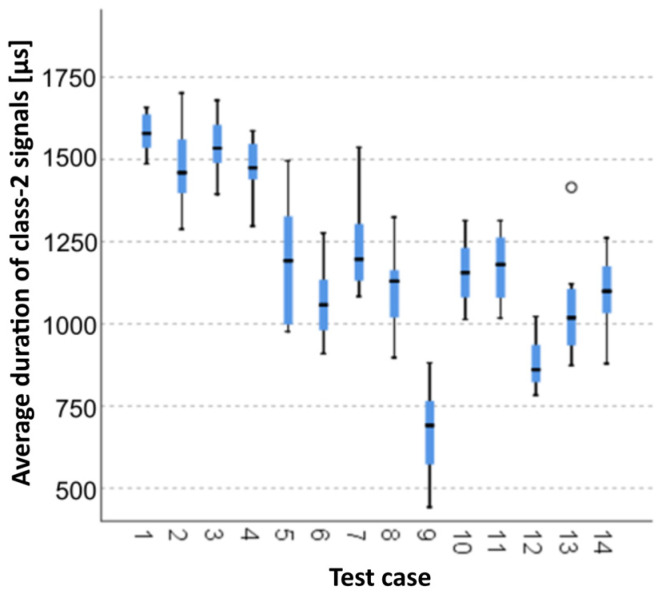
Graphic presentation of the Kruskal–Wallis test results for independent samples: average duration of class-2 signals.

The graphical representation of Kruskal–Wallis test results for samples of the average duration of class-2 signals (Figure 7) indicates that the longest average duration of class-2 signals was recorded for components from test case F_2_ (specimens soaked in water for 1 h). The largest scatter of results occurred for group F_5_ (specimens exposed to 50 bath-drying cycles). Test case F_13_ (specimens ignited for 10 min) contains individual outliers. The shortest average duration of class-2 signals was recorded for case F_9_ (specimens exposed to 100 freeze–thaw cycles).

#### 3.1.7. Number of Class-3 Signals

**Figure 8:**
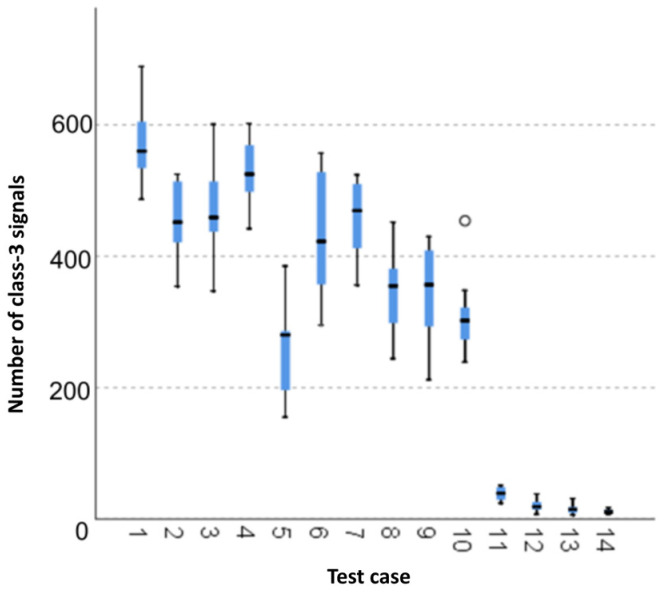
Graphic presentation of the Kruskal–Wallis test results for independent samples: number of class-3 signals.

The graphical representation of Kruskal–Wallis test results for independent samples of the number of class-3 signals (Figure 8) indicates that the highest number of class-3 signals was recorded for components from test case F_1_ (air-dried specimens). The largest scatter of results occurred for group F_6_ (specimens exposed to 10 freeze–thaw cycles). Test case F_10_ (specimens ignited for 2.5 min) contains individual outliers. The smallest number of class-3 signals was recorded for case F_14_ (specimens fired in a furnace).

#### 3.1.8. Average Strength of Class-3 Signals [nVs]

**Figure 9:**
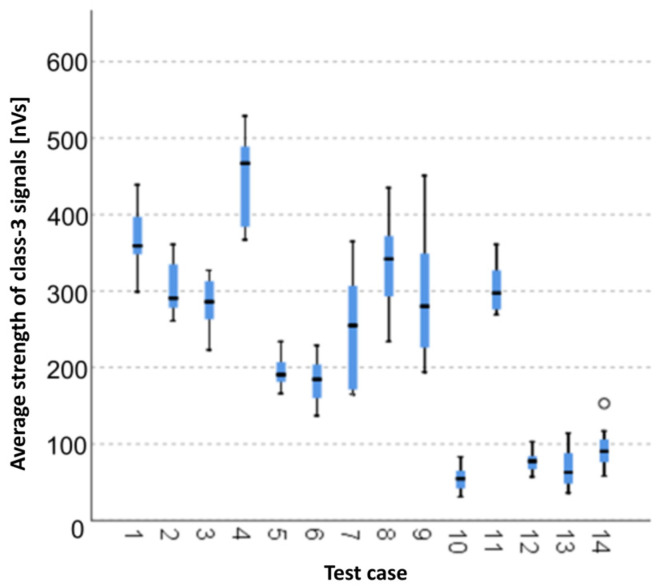
Graphic presentation of the Kruskal–Wallis test results for independent samples: average strength of class-3 signals.

The graphical representation of Kruskal–Wallis test results for independent samples of the average strength of class-3 signals (Figure 9) indicates that the highest average strength of class-3 signals was recorded for components from test case F_4_ (specimens exposed to 25 bath-drying cycles). The largest scatter of results occurred for group F_9_ (specimens exposed to 100 freeze–thaw cycles). Test case F_14_ (specimens fired in a furnace) contains individual outliers. The smallest strength of class-3 signals was recorded for case F_10_ (specimens ignited for 2.5 min).

#### 3.1.9. Average Duration of Class-3 Signals [µs]

**Figure 10:**
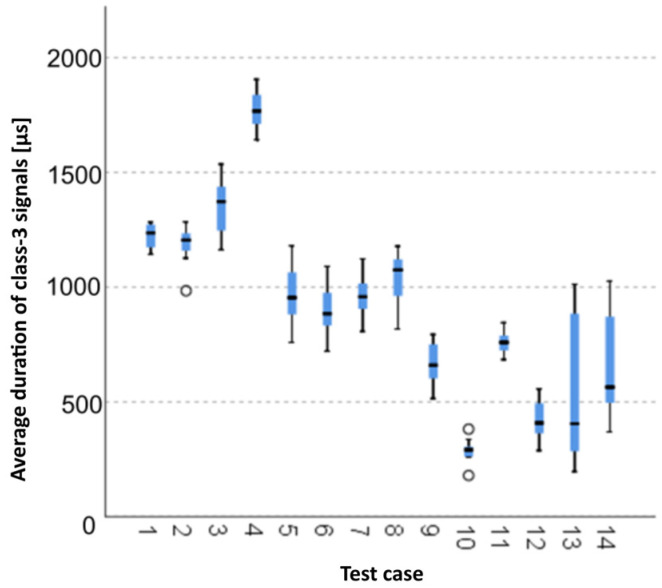
Graphic presentation of the Kruskal–Wallis test results for independent samples: average duration of class-3 signals.

The graphical representation of Kruskal–Wallis test results for independent samples of the average duration of class-3 signals (Figure 10) indicates that the longest average duration of class-3 signals was recorded for components from test case F_4_ (specimens exposed to 25 bath-drying cycles). The largest scatter of results occurred for group F_13_ (specimens ignited for 10 min). Test cases F_2_ (specimens soaked in water for 1 h) and F_10_ (specimens ignited for 5 min) contain individual outliers. The shortest average duration of class-3 signals was recorded for case F_13_ (specimens ignited for 10 min).

#### 3.1.10. Number of Class-4 Signals

**Figure 11:**
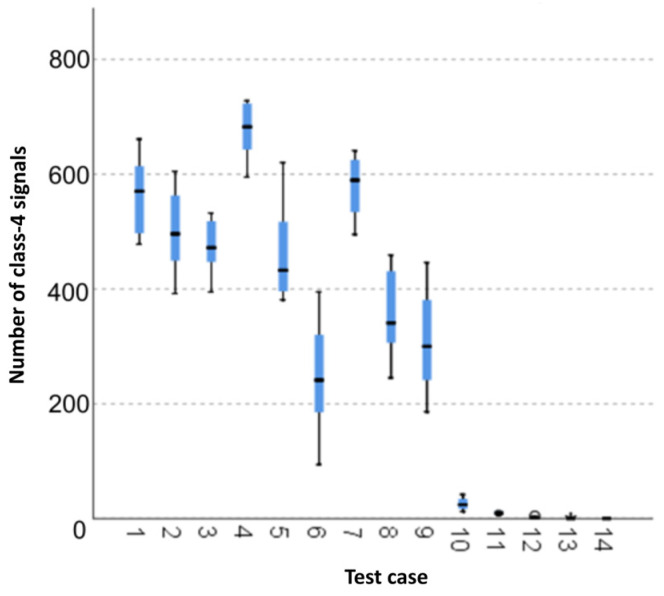
Graphic presentation of the Kruskal–Wallis test results for independent samples: number of class-4 signals.

The graphical representation of Kruskal–Wallis test results for independent samples of the number of class-4 signals (Figure 11) indicates that the highest number of class-4 signals was recorded for components from test case F_4_ (specimens exposed to 25 bath-drying cycles). The largest scatter of results occurred for group F_6_ (specimens exposed to 10 freeze–thaw cycles). The smallest number of class-4 signals was recorded for case F_14_ (specimens fired in a furnace).

#### 3.1.11. Average Strength of Class-4 Signals [nVs]

**Figure 12:**
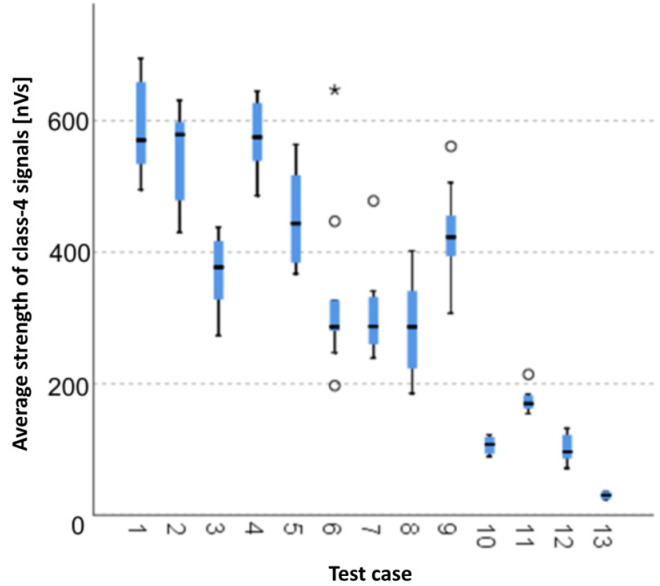
Graphic presentation of the Kruskal–Wallis test results for independent samples: average strength of class-4 signals.

The graphical representation of Kruskal–Wallis test results for samples of the average strength of class-4 signals (Figure 12) indicates that the highest average strength of class-4 signals was recorded for components from test case F_1_ (air-dried specimens). The largest scatter of results occurred for group F_8_ (specimens exposed to 50 freeze–thaw cycles). Test cases F_6_ (specimens exposed to 10 freeze–thaw cycles), F_7_ (specimens exposed to 25 freeze–thaw cycles), F_9_ (specimens exposed to 100 freeze–thaw cycles) and F_11_ (specimens ignited for 5 min) contain outliers. The smallest average strength of class-4 signals was recorded for case F_13_ (specimens ignited for 10 min).

#### 3.1.12. Average Duration of Class-4 Signals [µs]

**Figure 13:**
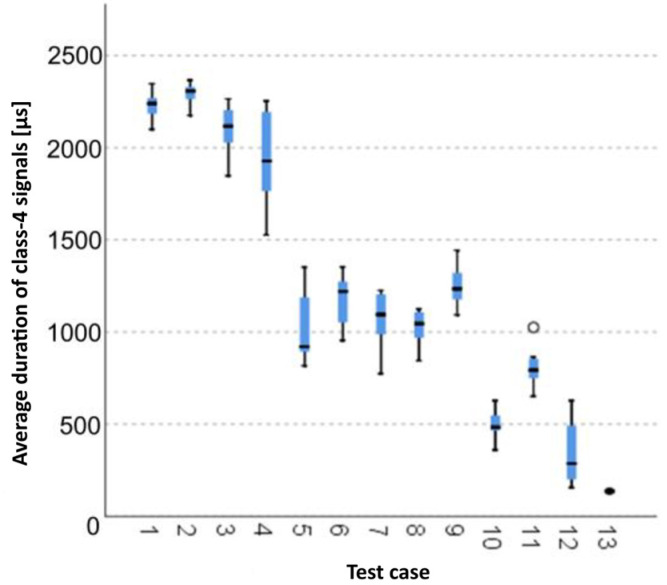
Graphic presentation of the Kruskal–Wallis test results for independent samples: average duration of class-4 signals.

The graphical representation of Kruskal–Wallis test results for independent samples of the average duration of class-4 signals (Figure 13) indicates that the longest average duration of class-4 signals was recorded for components from test case F_2_ (specimens soaked in water for 1 h). The largest scatter of results occurred for group F_4_ (specimens exposed to 25 bath-drying cycles). Test case F_11_ (specimens ignited for 5 min) contains individual outliers. The shortest average duration of class-4 signals was recorded for case F_13_ (specimens ignited for 10 min).

#### 3.1.13. Average Frequency of AE Events before Reaching F_max_ [kHz]

**Figure 14:**
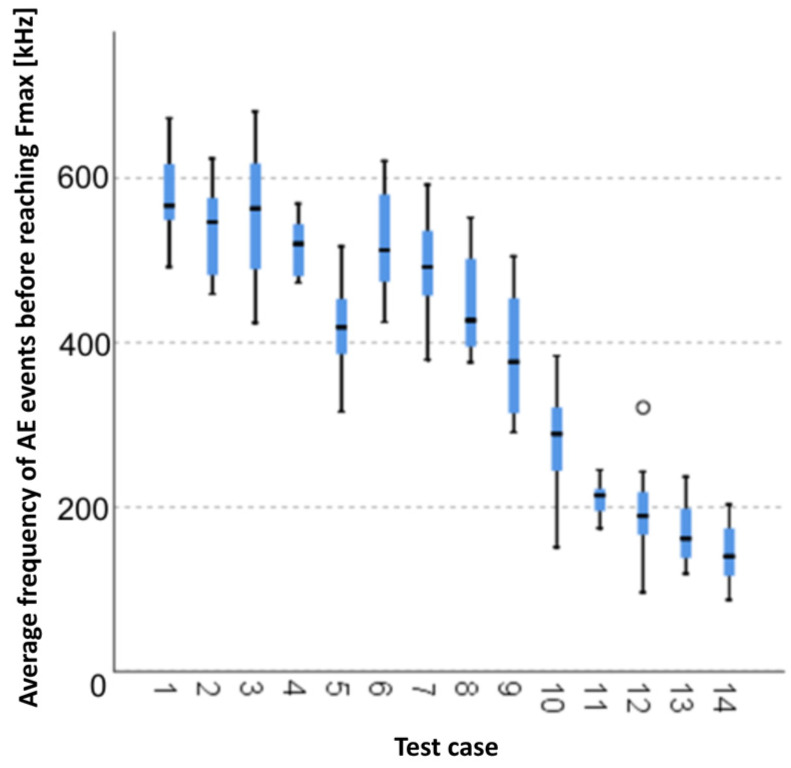
Graphic presentation of the Kruskal–Wallis test results for independent samples: average frequency of AE events before reaching F_max_.

The graphical representation of Kruskal–Wallis test results for independent samples of the average frequency of AE events before reaching F_max_ (Figure 14) indicates that the longest average frequencies were recorded for components from test case F_3_ (specimens soaked in water for 24 h). Moreover, for this case, the scatter of the results was the greatest. Test case F_12_ (specimens ignited for 7.5 min) contains individual outliers. The smallest average frequencies of AE events before reaching F_max_ were recorded for case F_14_ (specimens fired in a furnace).

#### 3.1.14. *MOR* Bending Strength [MPa]

**Figure 15:**
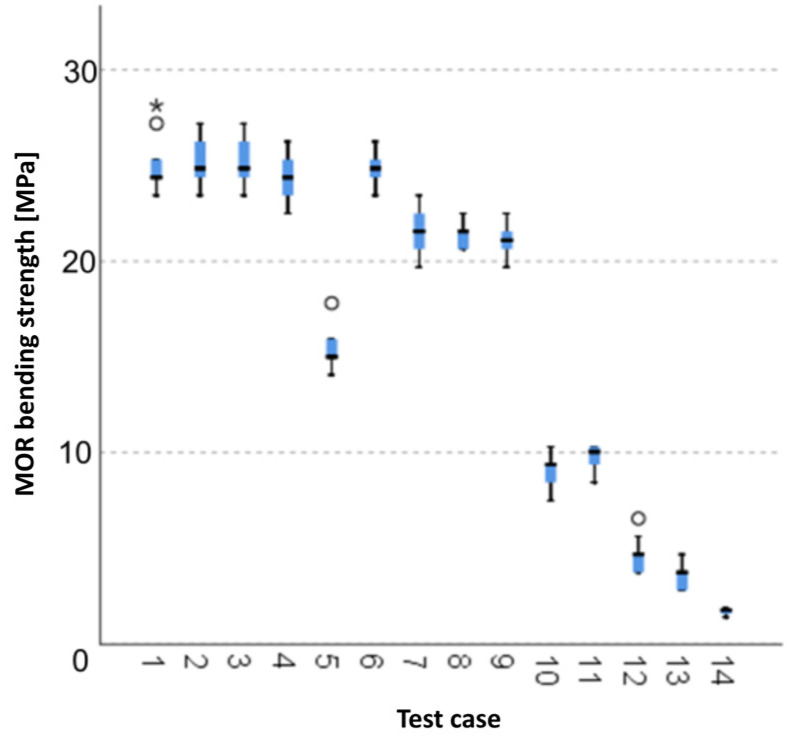
Graphic presentation of the Kruskal–Wallis test results for independent samples: *MOR* bending strength.

The graphical representation of Kruskal–Wallis test results for independent samples of the *MOR* bending strength (Figure 15) indicates that the highest strength was recorded for components from test case F_3_ (specimens soaked in water for 24 h). Moreover, for this case, the scatter of the results was the greatest. Test cases F_1_ (air-dried specimens), F_5_ (specimens exposed to 50 bath-drying cycles) and F_12_ (specimens ignited for 7.5 min) contain individual outliers. The smallest *MOR* bending strength was recorded for case F_14_ (specimens fired in a furnace).

### 3.2. Breakdown of the Number of Signals and Selected Energy Parameters into Groups Depending on the Significance of Changes

Classification trees using the CHAID algorithm were used to identify the divisions into signal characteristics.

#### 3.2.1. Number of Class-1 Signals

**Figure 16:**
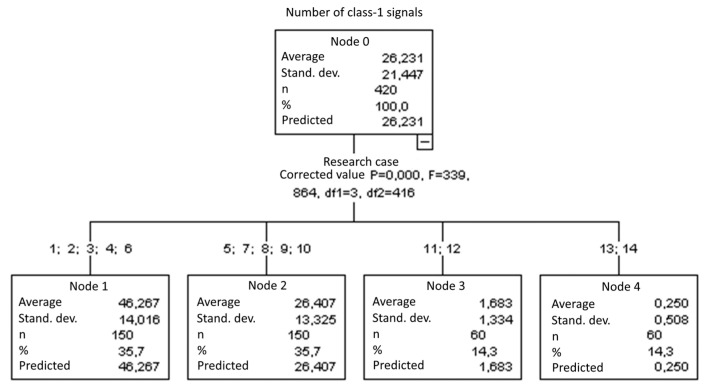
Classification tree for the number of class-1 signals.

Four groups were identified. For every successive group, there is a significant drop in the number of class-1 signals (Figure 16):

- Groups 1, 2, 3, 4 and 6;
- Groups 5, 7, 8, 9 and 10;
- Groups 11 and 12;
- Groups 13 and 14.

#### 3.2.2. Average Strength of Class-1 Signals [nVs]

**Figure 17:**
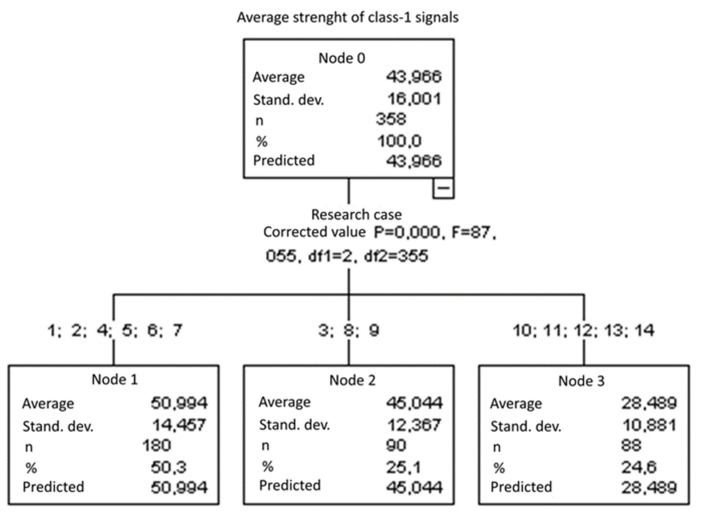
Classification tree for the average strength of class-1 signals.

Three groups were identified. For every successive group, there is a significant drop in the average strength of class-1 signals (Figure 17):

- Groups 1, 2, 4, 5, 6 and 7;
- Groups 3, 8 and 9;
- Groups 10, 11, 12, 13 and 14.

#### 3.2.3. Average Duration of Class-1 Signals [µs]

**Figure 18:**
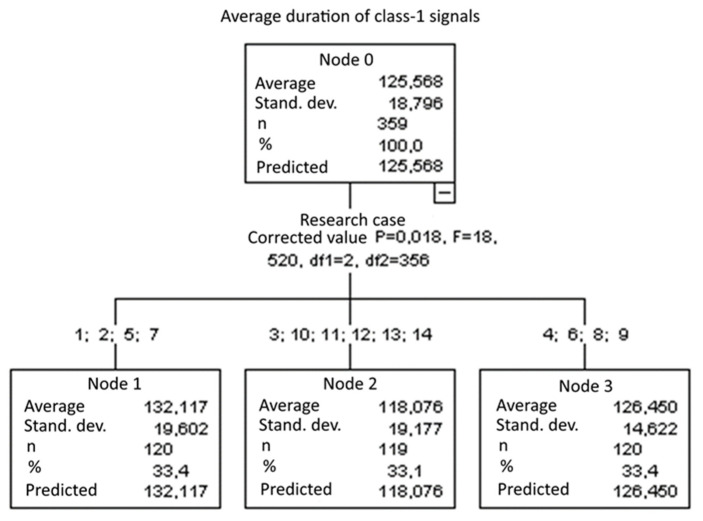
Classification tree for the average duration of class-1 signals.

Three groups were identified. For every successive group, there is a significant drop in the average duration of class-1 signals (Figure 18):

- Groups 1, 2, 5 and 7;
- Groups 4, 6, 8 and 9;
- Groups 3, 10, 11, 12, 13 and 14.

#### 3.2.4. Number of Class-2 Signals

**Figure 19:**
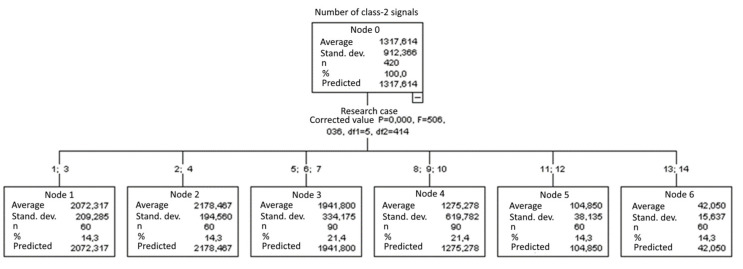
Classification tree for the number of class-2 signals.

Six groups were identified. For every successive group, there is a significant drop in the number of class-2 signals (Figure 19):

- Groups 2 and 4;
- Groups 1 and 3;
- Groups 5, 6 and 7;
- Groups 8, 9 and 10;
- Groups 13 and 14.

#### 3.2.5. Average Strength of Class-2 Signals [nVs]

**Figure 20:**
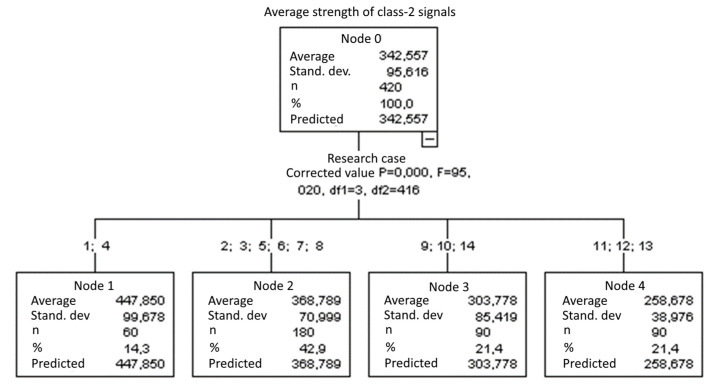
Classification tree for the average strength of class-2 signals.

Four groups were identified. For every successive group, there is a significant drop in the average strength of class-2 signals (Figure 20):

- Groups 1 and 4;
- Groups 2, 3, 5, 6, 7 and 8;
- Groups 9, 10 and 14;
- Groups 11, 12 and 13.

#### 3.2.6. Average Duration of Class-2 Signals [µs]

**Figure 21:**
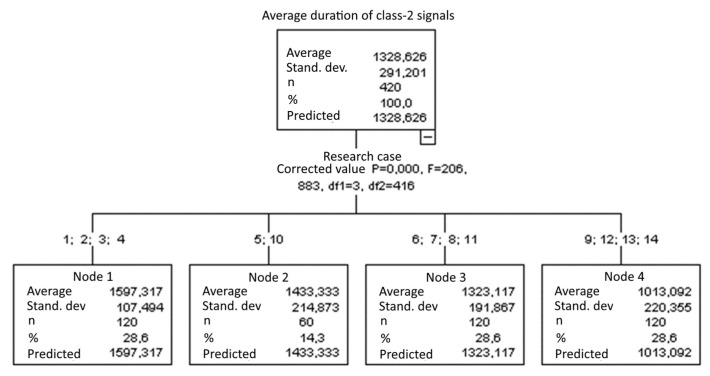
Classification tree for the average duration of class-2 signals.

Four groups were identified. For every successive group, there is a significant drop in the average duration of class-2 signals (Figure 21):

- Groups 1, 2, 3 and 4;
- Groups 5 and 10;
- Groups 6, 7, 8 and 11;
- Groups 9, 12, 13 and 14.

#### 3.2.7. Number of Class-3 Signals

**Figure 22:**
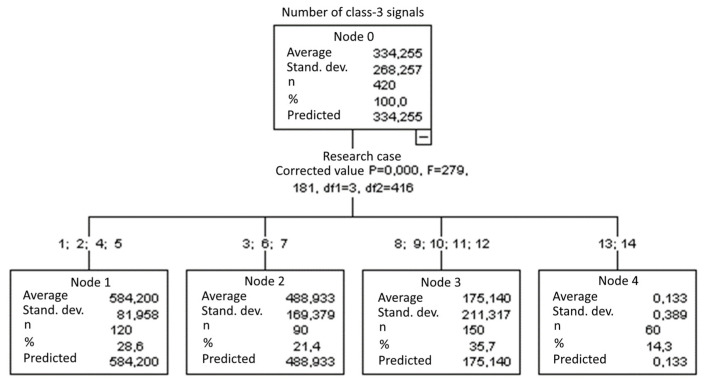
Classification tree for the number of class-3 signals.

Four groups were identified. For every successive group, there is a significant drop in the number of class-3 signals (Figure 22):

- Groups 1, 2, 4 and 5;
- Groups 3, 6 and 7;
- Groups 8, 9, 10, 11 and 12;
- Groups 13 and 14.

#### 3.2.8. Average Strength of Class-3 Signals [nVs]

**Figure 23:**
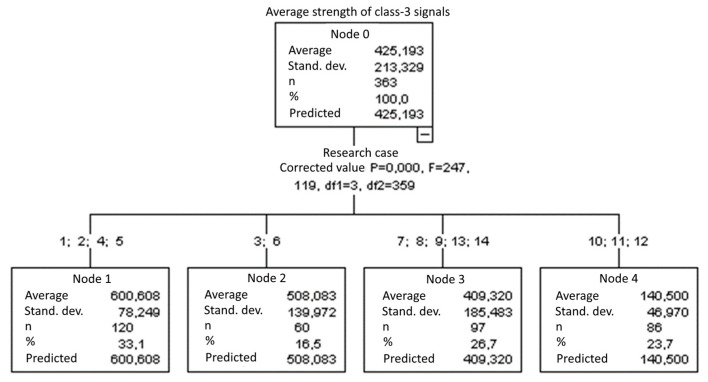
Classification tree for the average strength of class-3 signals.

Four groups were identified. For every successive group, there is a significant drop in the average strength of class-3 signals (Figure 23):

- Groups 1, 2, 4 and 5;
- Groups 3 and 6;
- Groups 7, 8, 9, 13 and 14;
- Groups 10, 11 and 12.

#### 3.2.9. Average Duration of Class-3 Signals [µs]

**Figure 24:**
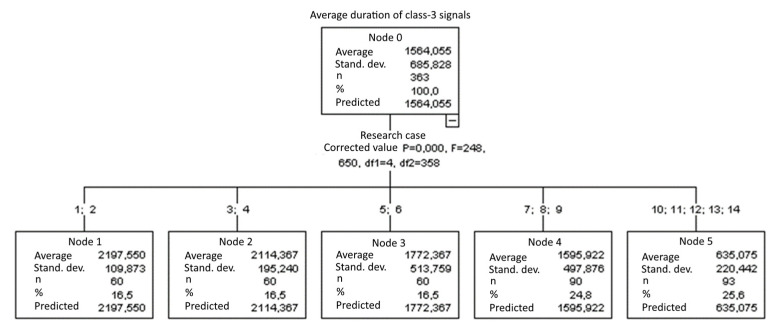
Classification tree for the average duration of class-3 signals.

Five groups were identified. For every successive group, there is a significant drop in the average duration of class-3 signals (Figure 24):

- Groups 1 and 2;
- Groups 3 and 4;
- Groups 5 and 6;
- Groups 7, 8 and 9;
- Groups 10, 11, 12, 13 and 14.

#### 3.2.10. Number of Class-4 Signals

**Figure 25:**
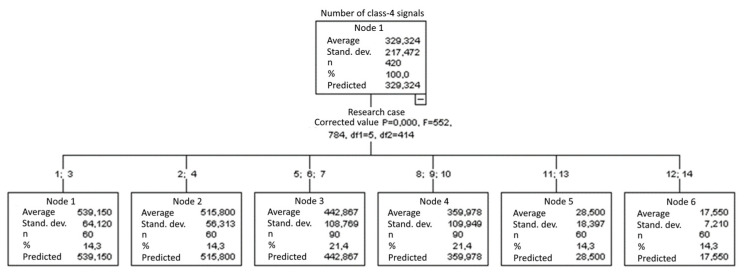
Classification tree for the number of class-4 signals.

Six groups were identified. For every successive group, there is a significant drop in the number of class-4 signals (Figure 25):

- Groups 1 and 3;
- Groups 2 and 4;
- Groups 5, 6 and 7;
- Groups 8, 9 and 10;
- Groups 11 and 13;
- Groups 12 and 14.

#### 3.2.11. Average Strength of Class-4 Signals [nVs]

**Figure 26:**
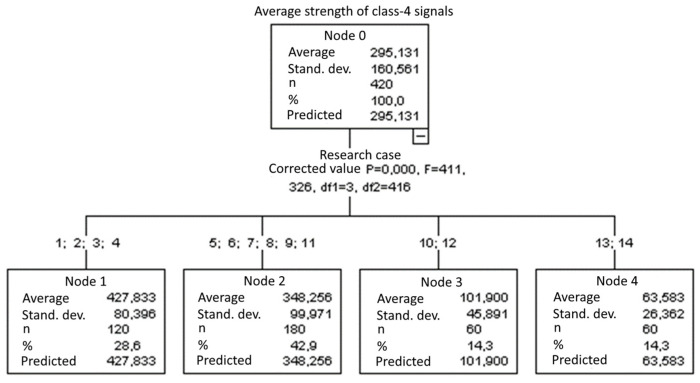
Classification tree for the average strength of class-4 signals.

Four groups were identified. For every successive group, there is a significant drop in the average strength of class-4 signals (Figure 26):

- Groups 1, 2, 3 and 4;
- Groups 5, 6, 7, 8, 9 and 11;
- Groups 10 and 12;
- Groups 13 and 14.

#### 3.2.12. Average Duration of Class-4 Signals [µs]

**Figure 27:**
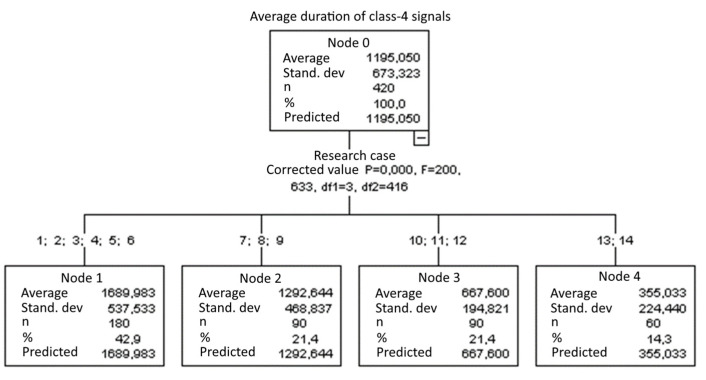
Classification tree for the average duration of class-4 signals.

Four groups were identified. For every successive group, there is a significant drop in the average duration of class-4 signals (Figure 27):

- Groups 1, 2, 3, 4, 5 and 6;
- Groups 7, 8 and 9;
- Groups 10, 11 and 12;
- Groups 13 and 14

#### 3.2.13. Average Frequency of AE Events before Reaching F_max_ [kHz]

**Figure 28:**
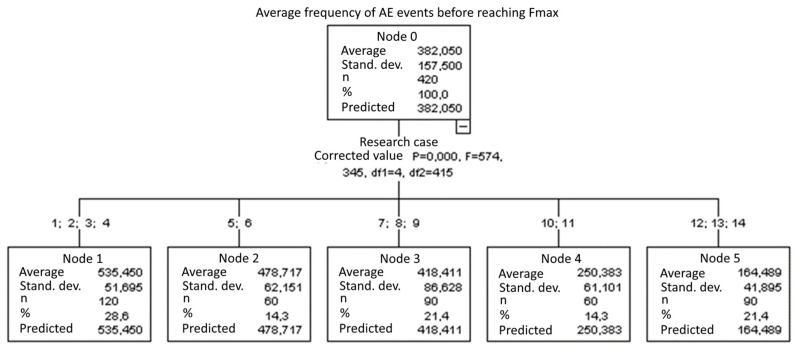
Classification tree for the average frequency of AE events before reaching F_max_.

Five groups were identified. For every successive group, there is a significant drop in the average duration of AE events (Figure 28):

- Groups 1, 2, 3 and 4;
- Groups 5 and 6;
- Groups 7, 8 and 9;
- Groups 10 and 11;
- Groups 12, 13 and 14.

#### 3.2.14. *MOR* Bending Strength [MPa]

**Figure 29:**
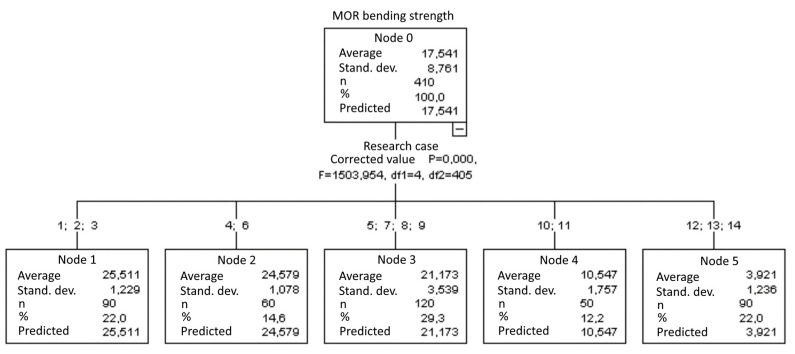
Classification tree for *MOR* bending strength.

Five groups were identified. For every successive group, there is a significant drop in the average duration of AE events (Figure 29):

- Groups 1, 2, 3 and 4;
- Groups 5 and 6;
- Groups 7, 8 and 9;
- Groups 10 and 11;
- Groups 12, 13 and 14.

### 3.3. Correlation between Parameters

In order to test the normality of the data distribution, the Shapiro–Wilk test was performed. In most cases, the data are not normally distributed. Therefore, the Spearman correlation coefficient was used to investigate the relationship between the indicated changes.

It was found that a significant correlation exists between:

- The number of class-1 signals and the bending strength *MOR* (*ρ* = 0.874, *p* = 0.000): the more signals there are, the higher the *MOR* bending strength.
- Average strength of class-1 signals and bending strength *MOR* (*ρ* = 0.581, *p* = 0.000): the higher the average signal strength is, the higher the *MOR* bending strength.
- The number of class-2 signals and the bending strength *MOR* (*ρ* = 0.844, *p* = 0.000): the more signals there are, the higher the *MOR* bending strength.
- Average strength of class-2 signals and bending strength *MOR* (*ρ* = 0.483, *p* = 0.000): the higher the average signal strength is, the higher the *MOR* bending strength.
- Average duration of class-2 signals and bending strength *MOR* (*ρ* = 0.548, *p* = 0.000): the greater the mean duration of the signals is, the higher the *MOR* bending strength.
- The number of class-3 signals and the bending strength *MOR* (*ρ* = 0.772, *p* = 0.000): the more signals there are, the higher the *MOR* bending strength.
- Average strength of class-3 signals and bending strength *MOR* (*ρ* = 0.690, *p* = 0.000): the higher the average signal strength is, the higher the *MOR* bending strength.
- Average duration of class-3 signals and bending strength *MOR* (*ρ* = 0.859, *p* = 0.000): the greater the mean duration of the signals is, the higher the *MOR* bending strength.
- The number of class-4 signals and the bending strength *MOR* (*ρ* = 0.847, *p* = 0.000): the more signals there are, the higher the *MOR* bending strength.
- Average strength of class-4 signals and bending strength *MOR* (*ρ* = 0.661, *p* = 0.000): the higher the average signal strength is, the higher the *MOR* bending strength.
- Average duration of class-4 signals and bending strength *MOR* (*ρ* = 0.750, *p* = 0.000): the greater the mean duration of the signals is, the higher the *MOR* bending strength.
- Average frequency of AE events before reaching F_max_ and the bending strength *MOR* (*ρ* = 0.875, *p* = 0.000): the higher the frequency of AE events is, the higher the *MOR* bending strength.

## 4. Discussion

The test results shown above indicate that the operating conditions affect the mechanical parameters of fibre–cement boards exposed to loads. The impact of moisture and low and high temperature significantly reduces the strength of fibre–cement boards in comparison with air-dried objects under optimum humidity conditions. In most cases, the strength parameters decrease with the increase in the test case number. The highest strength decrease was observed in specimens exposed to flame and high temperature, which are destroyed by the action of high temperature due to the nature of the fibres.

The behaviour of fibre–cement boards under load is connected with the presence of the acoustic emission phenomenon. The decrease in the strength parameters in the objects results in a change in the acoustic characteristics and the number of signals in the individual classes. The graphical representation of the Kruskal–Wallis test results indicates that the *MOR* bending strength for specimens exposed to fire and high temperature is more than 50% lower than for air-dried specimens and specimens exposed to water. The increased number of freeze–thaw cycles also has an impact on the strength of the specimens. Components exposed to more than 10 freeze–thaw cycles had a strength more than 30% smaller than the reference specimens soaked in water and exposed to bath-drying cycles. A similar dependency was indicated by the number of signals of the individual classes, their energy parameters and frequencies. The number, strength, duration and frequency also decrease along with the increase in the test case number.

The acoustic emission parameters that are most sensitive to the reduction in the mechanical parameters of the boards are the average strength and the average duration of class 3 signals, the average strength and the average duration of the class 4 signals and the average frequency of events before reaching the maximum load. The occurrence of class 3 signals with an average strength of signals in the range from 200 to 400 nVs and a duration in the range of 1000–1500 µs and of class 4 signals with an average strength in the range of 100 to 300 nVs and a duration in the range of 100–150 µs at average event frequencies above 200 kHz, but not higher than 300 kHz, is associated with a reduction in the strength of the boards from 30% to 50%. The occurrence of class 3 signals with an average strength of signals below 200 nVs and duration less than 1000 µs and the occurrence of class 4 signals with an average strength of less than 100 nVs and with a duration of less than 100 µs with an average frequency of events below 200 kHz is associated with a reduction in the strength of the boards by more than 50%.

## 5. Conclusions

The research of fibre–cement boards, so far, has focused mainly on the influence of operational factors and high temperatures on the boards, determined by the examination of the physicochemical parameters-mainly on the flexural strength (*MOR*). The literature describes only a few cases of testing fibre–cement boards using non-destructive methods, including the acoustic emission method. There are results available that have tried to apply the acoustic emission method to determine the effect of cellulose fibers on the strength of fibre–cement boards and distinguish the EA events emitted by the fibers from those emitted by the cement matrix. Research has shown that this method is suitable for testing fibre–cement boards. Attempts were also made to use the acoustic emission method to study the effect of fire and high temperatures on fibre–cement boards, where it was proposed to combine the acoustic emission method with artificial intelligence, including artificial neural networks (ANN). These studies confirmed the effectiveness of using the acoustic emission method to monitor the condition of fibre–cement boards, however, the issue of the statistical correlation of mechanical and acoustic parameters was not discussed.

The following conclusions can be drawn based on the results shown above:

- During the three-point bending of fibre–cement specimens, various material damage mechanisms occur.
- Some conditions that may appear during the use of fibre–cement components have a significant adverse effect on their strength parameters.
- The reduction in strength parameters is strictly connected with a change of acoustic parameters recorded for a material under load.
- The highest decrease in strength is connected with the most significant reduction in the number of signals of the individual classes, their strength, duration and frequency.
- The highest decrease in the strength, number and parameters of AE signals was observed for the specimens exposed to fire and high temperature.
- After analysing the results of the research, it was found that the acoustic emission method was suitable to be used for the monitoring and diagnostics of fibre–cement boards under load.

Due to the existence of a significant correlation between the mechanical parameters of fibre–cement boards and the number and parameters of acoustic emission signals of individual classes, according to the authors, further research should be undertaken in order to describe the specific ranges of the parameters of acoustic emission signals of individual classes, which would enable conclusions about the condition of fibre–cement boards under a low load value. The authors also plan to include the ultrasound method in the applied methodology, which would enable the detection of possible material voids and discontinuities in the distribution of reinforcing fibers.

## Data Availability

Not applicable.
